# Excessive gestational weight gain in accordance with the IOM criteria and the risk of hypertensive disorders of pregnancy: a meta-analysis

**DOI:** 10.1186/s12884-018-1922-y

**Published:** 2018-07-04

**Authors:** Min Ren, Hanying Li, Wei Cai, Xiulong Niu, Wenjie Ji, Zhuoli Zhang, Jianmin Niu, Xin Zhou, Yuming Li

**Affiliations:** 1 Graduate School of Medicine, Tianjin Medical University, Tianjin, 300070 China; 2 Tianjin Key Laboratory of Cardiovascular Remodeling and Target Organ Injury, Pingjin Hospital Heart Center, 220 Cheng-Lin Road, Tianjin, 300162 China; 3 Cardiovascular Institute, Tianjin Chest Hospital, Tianjin, 300222 China; 4 Department of Radiology, Northwestern University Feinberg School of Medicine, Chicago, IL USA; 5 Shenzhen Maternity and Child Healthcare Hospital, Southern Medical University, Province, Shenzhen, 511400 Guangdong China

**Keywords:** Gestational weight gain, Hypertensive disorders of pregnancy, IOM recommendations, Meta-analysis

## Abstract

**Background:**

Excessive gestational weight gain (GWG) is a potential risk factor for hypertensive disorders of pregnancy (HDP).

**Methods:**

We systematically reviewed three electronic databases for relevant articles published in English: PubMed, EMBASE and Web of Science. The Newcastle-Ottawa Scale was used to assess study quality. Random-effects meta-analyses were performed to supply a pooled estimation of the *OR* comparing the risk of HDP among healthy pregnant women with and without excessive GWG.

**Results:**

The pooled estimation for the association between excessive GWG and the risk of HDPs yielded an odds ratio (OR) of 1.79 (95% CI: 1.61–1.99). A subgroup analysis showed that women who had excessive GWG were more likely to have an HDP (OR 1.82; 95% CI 1.53–2.17), preeclampsia (OR 1.92; 95% CI 1.36–2.72), or gestational hypertension (OR 1.67; 95% CI 1.43–1.95). The pooled estimation for the association between excessive GWG and the risk of HDPs among pregestational normal weight women yielded an OR of 1.57 (95% CI 1.26–1.96). A subgroup analysis showed that women who had excessive GWG were more likely to have HDP (OR 1.45; 95% CI 1.09–1.92) or gestational hypertension (OR 1.51; 95% CI 1.22–1.86). The summary ORs of pre-gestational underweight women and pre-gestational overweight and obese women were 2.17 (95% CI 1.56–3.02) and 1.32 (95% CI 1.08–1.63), respectively.

**Conclusions:**

The findings of this study suggest that excessive GWG in accordance with the IOM recommendations influences the rate of HDP.

**Electronic supplementary material:**

The online version of this article (10.1186/s12884-018-1922-y) contains supplementary material, which is available to authorized users.

## Background

The hypertensive disorders of pregnancy (HDP) including gestational hypertension (GH) and preeclampsia (PE) are major complications of pregnancy associated with increased risks of maternal and perinatal morbidity and mortality [1–3]. However, effective therapies for HDP remain limited, and women with HDPs are at risk for subsequent chronic hypertension and cardiovascular disease later in life [4–6]. HDP’ etiology is not completely understood; recent evidence suggests that prepregnancy body mass index (BMI) and excessive gestational weight gain (GWG) are modifiable factors associated with HDP [7].

GWG is a normal and expected component of a healthy pregnancy. This condition encompasses the uterus and its contents (i.e., fetus, amniotic fluid and placenta), plasma volume expansion, blood and interstitial fluid and maternal new fat and protein deposition [8–10]. However, excessive GWG is an independent and modifiable risk factor for the adverse complications of pregnancy [11]. In 1990, the Institute of Medicine (IOM) published guidelines for appropriate weight gain during pregnancy to support optimal optimum pregnancy outcomes [12]. In 2009, the IOM updated these guidelines for GWG to match the “dramatic shifts in the demographic and epidemiologic profile” of “U.S. women of childbearing age” [13]. The changes from the 1990 guidelines included a limited range of 5–9 kg for weight gain among obese women instead of the open-ended recommendation of 6.8 kg and a change in the classification parameters resulting in fewer women classified as underweight and more women classified as overweight. The recommended weight gains for underweight (BMI < 18.5 kg/m^2^), normal weight (BMI = 18.5–24.9 kg/m^2^), overweight (BMI = 25.0–29.9 kg/m^2^) and obese women (BMI > 30.0 kg/m^2^) were 12.5–18, 11.5–16, 7.0–11.5 and 5.0–9.0 kg, respectively.

However, few studies have examined the association between the current IOM guidelines and risk of HDP, and the results of current studies have inconsistently reported their outcomes. In this meta-analysis study, we evaluated the association between excessive GWG and the risk of HDP among healthy pregnant women based on the current 2009 IOM recommendations.

## Methods

### Search strategy

This review conforms to the Meta-Analysis of Observational Studies in Epidemiology (MOOSE) guidelines for reporting a meta-analysis on observational studies [14]. We searched the PubMed, EMBASE and Web of Science databases to identify relevant studies. We used the following search terms: (“gestation” OR “gestational” OR “pregnancy” OR “pregnant” OR “maternal” OR “prenatal”) and (“weight gain” OR “weight change” OR “weight increase”) and (“hypertension” OR “hypertension disorder complicating pregnancy” OR “hypertension disorder of pregnancy” OR “pregnancy-induced hypertension” OR “preeclampsia” OR “blood pressure”). Because only de-identified pooled data from individual studies, ethics approval was unnecessary for this study were analyzed.

### Inclusion and exclusion criteria

We included observational studies meeting the following inclusion criteria: (1) published in English; (2) published since 1990; (3) singleton pregnancies; (4) delivery at term (37–42 weeks); (5) reported an association between GWG and hypertension disorder of pregnancy, gestational hypertension or preeclampsia; (6) GWG was classified as above, within or below the Institute of Medicine (IOM) recommendations; (7) pre-pregnancy BMI was categorized as underweight, normal weight, overweight or obese in accordance with the classifications of the World Health Organization (WHO).

We excluded (1) studies of women with specific comorbid conditions; (2) studies of adolescent pregnancies; (3) studies that performed an intervention; (4) studies that did not report information pertinent to the key outcomes; (5) reviews, editorials, commentaries or letters to the editor and conference abstracts; (6) articles that described aspects of the same population.

#### Data abstraction and quality appraisal

Two researchers independently reviewed all identified abstracts and titles. Both researchers fully assessed the remaining articles. When an article was in dispute, a third researcher helped to determine a final decision. In addition, the reference lists of relevant and related articles were searched to ensure a complete literature. If a study reported different outcomes, then all outcomes were included in the meta-analysis.

Two reviewers extracted information independently using a standardized data collection form for all included studies. The third reviewer adjudicated any disagreement. For each study, we abstracted the following information: first author, year of publication, population information (i.e., country of origin, sample size, and gestational age at study entry), study characteristics (i.e., study design, definition of GWG, definition of outcomes, and inclusion/exclusion criteria), information about the outcome (i.e., the number of interesting outcomes and confounds). We measured the quality of the studies using the Newcastle-Ottawa Scale for assessing the cohort studies used in meta-analyses.

### Statistical analyses

Individual studies’ odds ratios (OR) and 95% confidence intervals (CI) were calculated based on the event numbers extracted from each study before data pooling. The heterogeneity among the studies was quantified and tested using the chi-square test and the *I*^*2*^ statistic, which represents the percentage of total variation across studies due to heterogeneity rather than chance. The assumption of homogeneity was considered invalid for *P*-values less than 0.10. *I*^*2*^ values of 25, 50 and 75% were regarded as low, moderate and high heterogeneity, respectively. A forest plot was generated for each analysis. When significant heterogeneity was found between studies, a random-effects model was employed.

### Sensitivity analysis and publication bias

Furthermore, we performed a sensitivity analysis by removing each study from the meta-analysis to investigate the influence of a single study on the overall effect. Potential publication bias was assessed with funnel plots, which charted the standard error of the studies against their corresponding size differences. In addition, Egger’s linear regression test and Begg’s rank correlation test were conducted to detect publication bias. All reported *P*-values were two-tailed, and those less than 0.05 were considered as significant unless otherwise specified. All statistical analyses were performed using STATA version 14.2 (STATA Corp., College Station, TX).

## Results

### Selection of studies

**Fig. 1:**
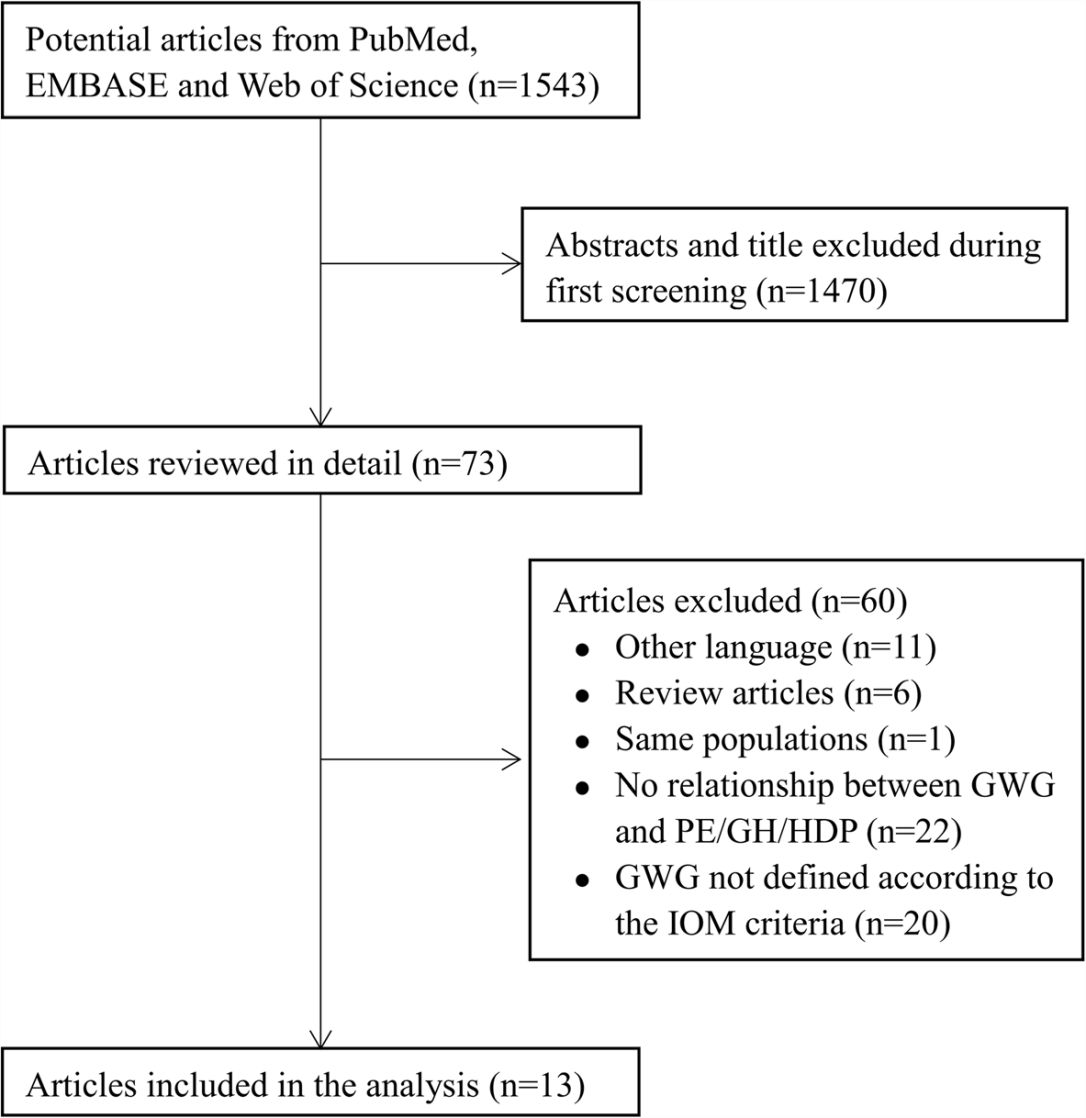
Selection of studies for the meta-analysis

We identified 1543 articles from our initial electronic search. Of these articles, 1470 were excluded after examining the abstract and title. The full texts of the remaining 73 articles were assessed for eligibility. Eleven articles were eliminated since they were not published in English, six were review articles, one article reported the same populations, twenty-two did not investigate the relationship between GWG and hypertension disorder of pregnancy or other related outcomes, and twenty did not classify weight gain according to the IOM criteria. Finally, thirteen articles [15–27] met our inclusion criteria and were included in the analysis (Fig. 1).

### Characteristics of studies included

The author group, type of cohort, sample size, definitions of GWG and outcomes, and inclusion/exclusion criteria were identified and are detailed in Additional file 1: Table S1 for the 13 articles included in the meta-analysis [15–27]. The 13 studies included a total of 156,170 participants. All included studies were cohort studies; seven were retrospective [15, 16, 18, 19, 24–26], three were prospective [20, 21, 23], two were retrospective analyses of prospectively collected data [17, 27] and one was a secondary analysis of a multicenter, placebo-controlled randomized double-blind trial [22]. Four studies included multiethnic populations [20, 22, 23, 27], seven studies were performed on Asian populations [15–19, 25, 26], one study was performed on a population of Latinas [21] and one study was performed on an African population [24].

Pre-pregnancy weight was self-reported in five studies [15–17, 21, 26] and measured at the first prenatal visit in five studies [18, 20, 22, 23, 25]; three studies did not mention their data-collection methods [19, 24, 27]. Seven studies with complete data for GWG with normal pre-gestational BMI used the IOM recommendations [15–17, 19, 23, 25, 27].

All studies included in the review were submitted to a methodological quality evaluation through the Newcastle-Ottawa Scale [28] (Table 1). Studies with scores of 0–4 or 5–8 were regarded as low and high quality, respectively. The scores ranged from 6 to 8.

**Table 1:** Quality assessment of the included studies using the Newcastle-Ottawa Scale

Author group	Selection				Comparability	Outcome			Total
	Representativeness	Non-exposed	Ascertainment	Outcome	Covariates	Assessment	Follow up	Lost to follow up	
Hung, 2016 [15]	☆	☆	☆	☆	☆	☆	☆	☆	8
Tanaka, 2014 [16]		☆	☆	☆	☆	☆	☆	☆	7
Liu, 2015 [17]	☆	☆	☆	☆	☆	☆	☆	☆	8
Li C,2015 [18]	☆	☆	☆	☆	☆	☆	☆	☆	8
Enomoto, 2016 [19]	☆	☆		☆	☆		☆	☆	6
Chung, 2013 [20]	☆	☆	☆	☆	☆	☆	☆		7
Chasan-Taber, 2016 [21]	☆	☆	☆	☆	☆	☆	☆	☆	8
Johnson, 2013 [22]	☆	☆	☆	☆	☆		☆	☆	7
Hannaford, 2017 [23]		☆	☆		☆	☆	☆	☆	6
Fouelifack, 2015 [24]	☆	☆	☆	☆		☆	☆	☆	7
Li N, 2013 [25]	☆	☆	☆	☆	☆	☆	☆	☆	8
Zhou, 2015 [26]	☆	☆		☆	☆		☆	☆	6
de la Torre L, 2011 [27]	☆	☆	☆	☆		☆	☆	☆	7

### Outcomes

**Fig. 2:**
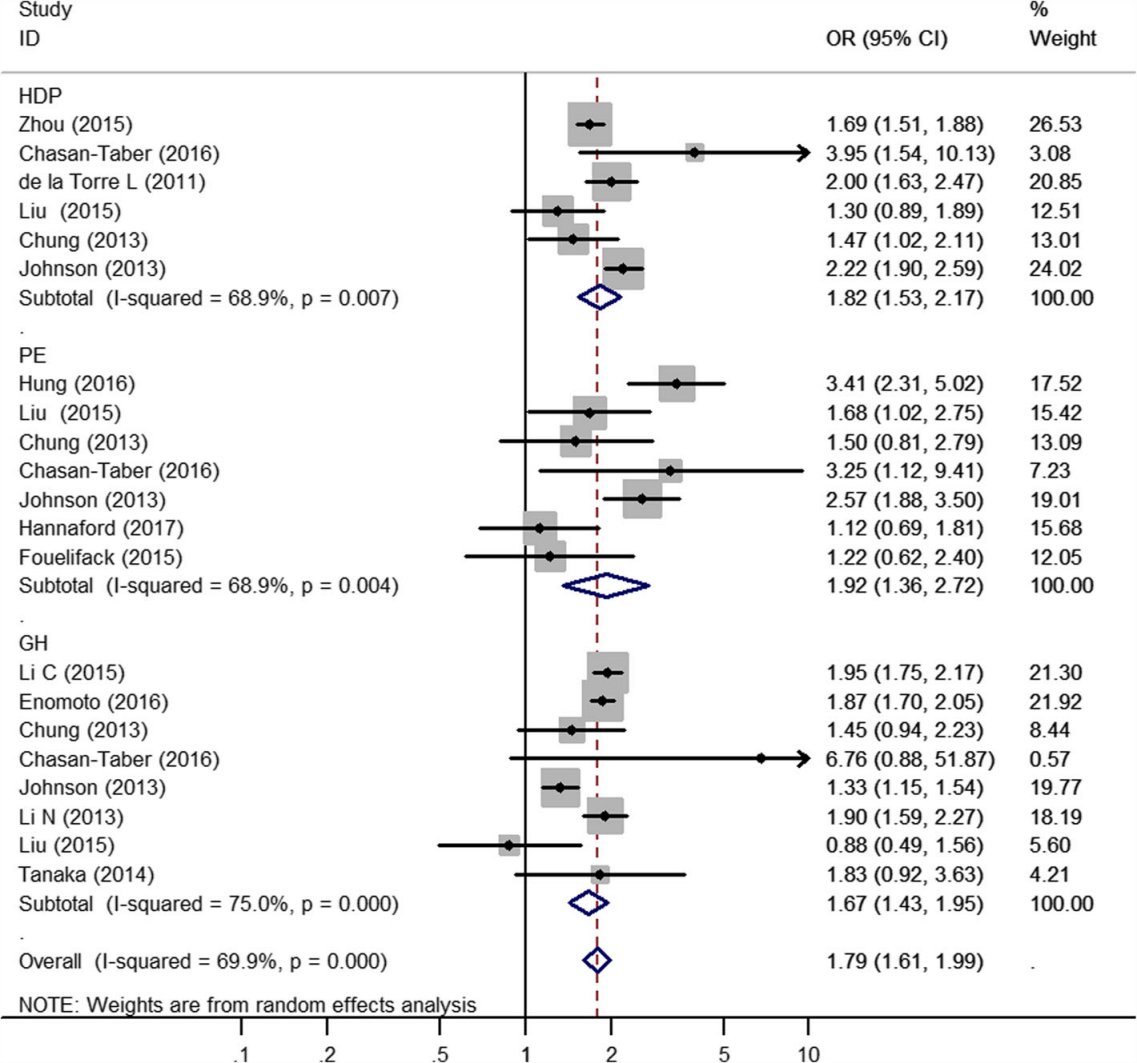
Forest plot of the random-effects model showing the pooled estimate of the odds of GWG above the IOM recommendations

The pooled results across the 21 trials included in the meta-analysis showed that GWGs above the IOM recommendations increased the risk of HDPs,the combined results included HDP, PE and GH, (unadjusted OR 1.79; 95% CI 1.61–1.99; Fig. 2). The between-study heterogeneity was moderate (*I*^*2*^ = 69.9%,*P* = 0.000). We also analyzed all subtypes of hypertension disorder of pregnancy in women with GWGs above the IOM recommendations. Seven studies with the outcome preeclampsia, eight studies with the outcome gestational hypertension and six studies with the outcome hypertension disorder of pregnancy were eligible for the meta-analysis. Significant heterogeneity was found between the studies for both subtypes (*I*^*2*^ = 68.9%; *P* = 0.004 for preeclampsia, *I*^*2*^ = 75.0%, *P* = 0.000 for gestational hypertension, and *I*^*2*^ = 68.9%, *P* = 0.007 for hypertension disorder of pregnancy), and random-effects models were employed for the meta-analysis. Women who gained more weight than recommended by the guidelines were more likely to have preeclampsia than those who gained weight within the guidelines (OR 1.92; 95% CI 1.36–2.72). Similar results were found with regard to gestational hypertension (OR 1.67; 95% CI 1.43–1.95) and HDP (OR 1.82; 95% CI 1.53–2.17). A sensitivity analysis that excluded one small weight gain study [21] did not change the significance. When excluded retrospective studies [15, 16, 18, 19, 24–26], the OR of GH decreases from 1.66 to 1.30 and the total OR decreases (Additional file 2: Figure S1).

**Fig. 3:**
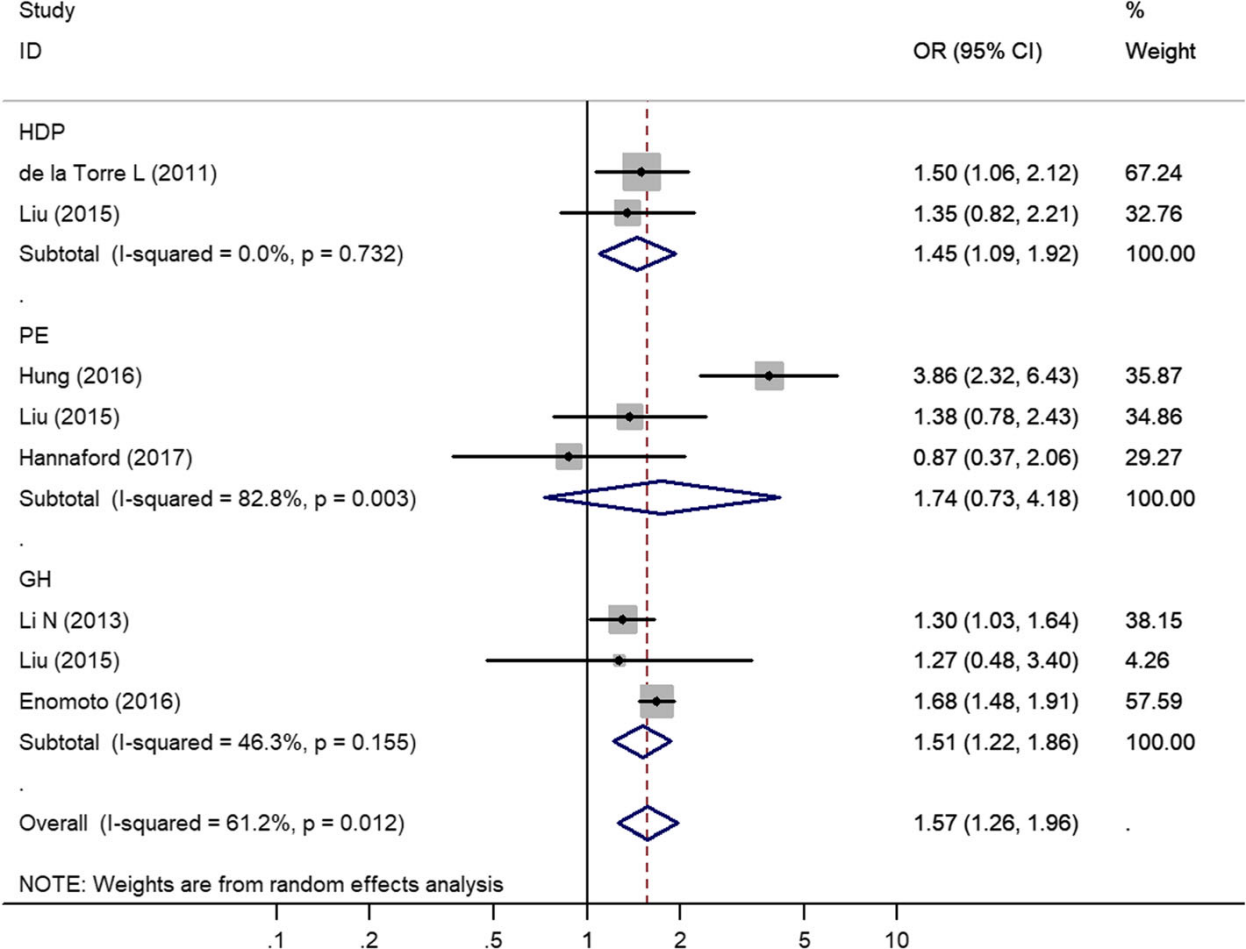
Forest plot of the random-effects model showing the pooled estimate of the odds of GWG above the IOM recommendations among women with pregestational normal weight

We analyzed the effects of excessive GWG during pregnancy on HDP in women with different pre-pregnancy BMI categories. The eight trials that were included in the meta-analysis showed that GWGs above the IOM recommendations increased the risk of HDPs among pre-gestational normal weight women (unadjusted OR 1.57; 95% CI 1.26–1.96; Fig. 3). The between-study heterogeneity was moderate (*I*^*2*^ = 61.2%, *P* = 0.012). We also analyzed all subtypes of hypertension disorder of pregnancy among pre-gestational normal weight women with GWGs above the IOM recommendations. Three studies with the outcome preeclampsia, three studies with the outcome gestational hypertension and two studies with the outcome hypertension disorder of pregnancy were eligible for the meta-analysis. Significant heterogeneity was found between the studies for preeclampsia, *I*^*2*^ = 82.8%; *P* = 0.003. No significant heterogeneity existed between the studies for gestational hypertension, *I*^*2*^ = 46.3%, *P* = 0.155, or for hypertension disorder of pregnancy, *I*^*2*^ = 0.0%, *P* = 0.732. Women who gained more weight than the guidelines recommended were more likely to have gestational hypertension than those who gained weight within the guidelines (OR 1.51; 95% CI 1.22–1.86), and similar results were found with regard to HDP (OR 1.45; 95% CI 1.09–1.92). No significant difference was found with regard to preeclampsia for GWGs above the IOM (OR 1.74; 95% CI 0.73–4.18). A sensitivity analysis excluding one small weight gain study [17] did not change the significance. When excluded retrospective studies [15, 19, 25]. There was no statistically significant difference when the OR of PE decrease, and the total OR was decreased (Additional file 3: Figure S2).

**Fig. 4:**
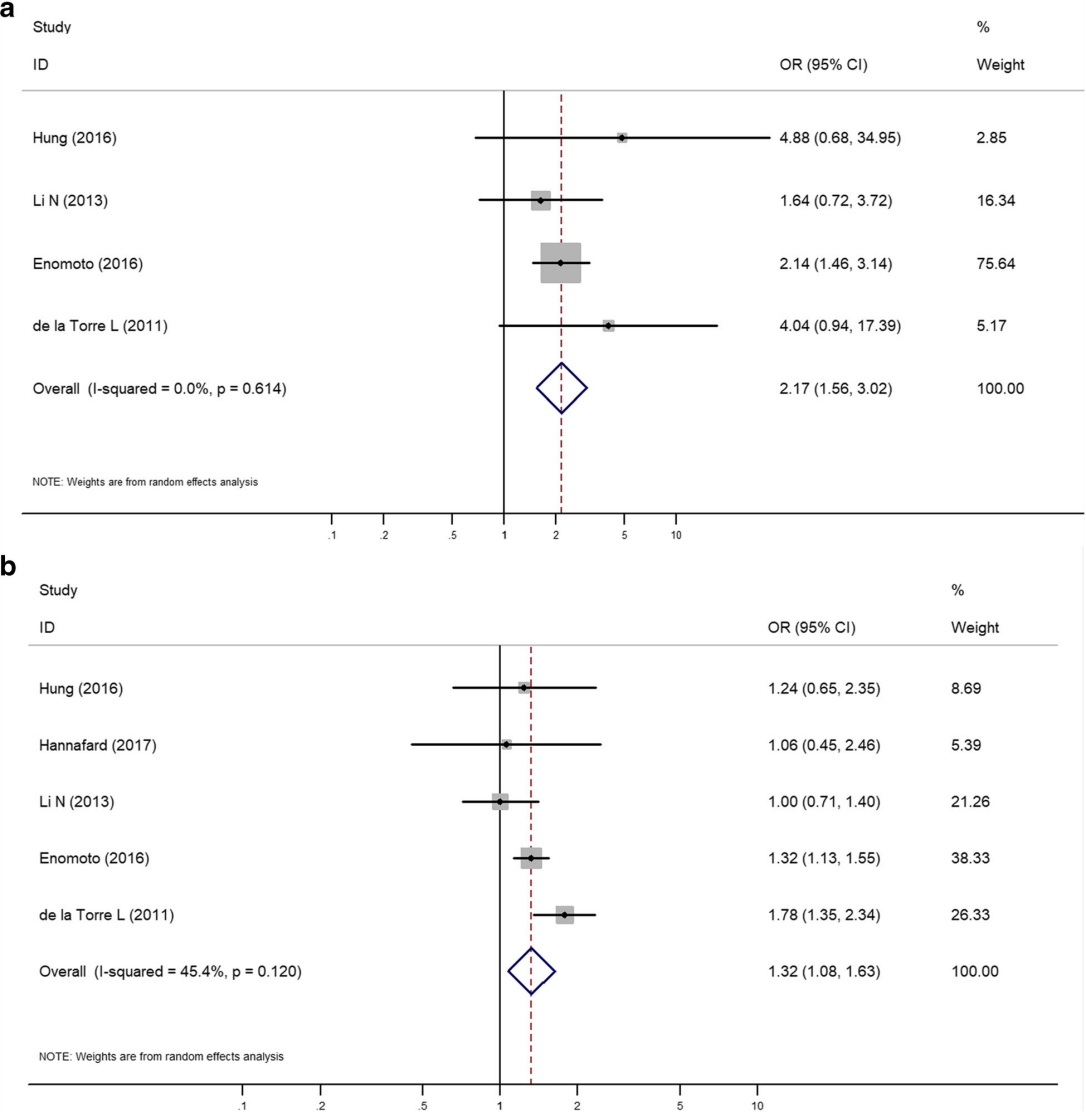
Forest plot of the random effects model showing the pooled estimate of the odds of GWG above the IOM recommendations among pregestational underweight women (**a**) and pregestational overweight and obese women (**b**); **a** the odds of GWG above the IOM recommendations among women with pregestational underweight weight; **b** The odds of GWG above the IOM recommendations among women with pregestational overweight and obese weight

Four studies of pre-gestational underweight women and five studies of pre-gestational overweight and obese women were included in this meta-analysis; the summary ORs were 2.17 (95% CI 1.56–3.02) and 1.32 (95% CI 1.08–1.63), respectively (Fig. 4).

Potential publication bias was observed using funnel plots. The funnel plot that showed a relationship between the odds ratios and the standard errors of the logarithmic odds ratios suggested no publication bias (Additional file 4: Figure S3). Egger’s and Begg’s tests yielded results that were similar to those of the funnel plots (above vs. within; Egger’s *P* = 0.766; Begg’s *P* = 0.695).

## Discussion

Over recent decades, HDP has remained a leading cause of maternal and fetal mortality and morbidity worldwide. The 2009 IOM guidelines suggested that “while the relationship between overweight/obese BMI and the rates of hypertension is shown in the literature, the relationship with high GWG requires more studies” [13]. Although several previous studies have implied that excessive weight gain throughout pregnancy poses an important risk for producing HDP [27, 29–31], this conclusion has not been confirmed due to the overall restricted number of researches and the associated study’s potential design limitations. In this meta-analysis, we examined the associations between excessive GWG and the risk of HDP among healthy pregnant women who reported their GWGs based on the current 2009 IOM recommendations and assessed the risk of HDP among women with normal pre-gestational BMIs. We found that women with excessive GWG above the IOM guidelines were nearly 1.8 time more likely to have HDP than those who gained weight within the guidelines.

The present study evaluated GWG as the change throughout pregnancy and did not separate weight gain owning to edema from weight gain because of adiposity. Although edema also occurs in normotensive pregnancies [32], it is more likely to occur in women who develop preeclampsia, which might result in greater weight gain during pregnancy and a consequent overestimation of excessive GWG on the odds of PE. It is difficult to distinguish whether increased edema in women with preeclampsia causes greater weight gain or whether greater weight gain influences preeclampsia. Women with gestational hypertension should be less likely to have edema [1] because this condition is not characterized based on proteinuria; therefore, the influence of weight gain is more likely through adiposity. To eliminate this influence, we individually assessed the risks of preeclampsia and gestational hypertension. In our study, the odds of preeclampsia and gestational hypertension were 1.92 (95% CI 1.36–2.72) and 1.67 (95% CI 1.43–1.95), respectively. The odds of HDP, which includes preeclampsia and gestational hypertension, was 1.82 (95% CI 1.53–2.17).

Because edema is unlikely to occur during early pregnancy, the association between excessive weight gain at this stage of pregnancy and the risk of HDP is unlikely to be explained by edema, which suggests that GWG precedes the development of HDP. However, studies of weight gain during early pregnancy and the risk of HDP are sparse. The Avon Longitudinal Study of Parents and Children (ALSPAL) [29] attempted to determine whether weight gain during early pregnancy is a risk factor for preeclampsia and gestational hypertension. They found that excessive weight gain during early pregnancy (up to 18 weeks) was independently associated with increased risks of developing preeclampsia and gestational hypertension after adjusting for pre-pregnancy weight (per 200 g/wk. increase in GWG up to 18 weeks: OR 1.31; 95% CI 1.07–1.62 and OR 1.26; 95% CI 1.16–1.38, respectively). Our study included fewer articles about early pregnancy, and no statistical analyses could be performed. More research is needed to confirm the causal relationship between weight gain during early pregnancy and the risk of HDP.

Recent evidence suggests that excessive GWG and elevated pre-pregnancy BMI are important factors for HDP. To evaluate the individual role of GWG, we analyzed the effects of excessive GWG during pregnancy on HDP among women with different pre-pregnancy BMI categories. Our study found that GWGs relative to the IOM guidelines showed differential effects on the rates of HDP among women of different pre-pregnancy weight categories. The pooled analysis of the unadjusted OR of HDP yielded a summary OR of 1.57 (95% CI 1.26–1.96) for pre-pregnancy normal weight women, 2.17 (95% CI 1.56–3.02) for pre-pregnancy underweight women and 1.32 (95% CI 1.08–1.63) for pre-pregnancy overweight and obese women. Unfortunately, only four of the involved studies provided supplied additional data on GWG stratified by pre-pregnancy BMI. More research is needed to investigate the individual role of GWG.

Several limitations of our study merit attention. First, using self-reported pre-pregnancy weight or the weight and height data recorded at the first prenatal visit within the first 12 weeks of pregnancy is one limitation of this study. In our report, pre-pregnancy weight was self-reported by five studies [15–17, 21, 26] and measured at the first prenatal visit by five studies [18, 20, 22, 23, 25]; three studies did not mention their data collection methods [19, 24, 27]. Self-reported pre-pregnancy weight is subject to recall bias, and the weight recorded at the first prenatal visit is difference from pre-pregnancy weight, which might lead to under- or overestimations of GWG, a common problem among gestational weight gain studies. Nevertheless, self-reported pre-gravid weight has been shown to align fairly closely with measured weights [33] Oken et al. recently reported a general correlation coefficient of 0.99 between self-reported and measured pre-gravid weight [34]. Although the best approach for assessing weight gain throughout pregnancy is based on entered weight at conception, these data were not available.

Second, the IOM recommendations are based on population information from North America, which restricts its use among populations with different ethnicities. Because Asian women generally have a lower BMI prior to pregnancy than those in Western countries, the BMI criteria developed by the WHO are not suitable for Asian populations, which might result in differences between Asian and Western populations. These hypotheses have not been extensively studied. However, no recommendations exist for new and clear BMI cut-off points among Asians. Only two of the included articles used Asian standards.

Lastly, because the included studies are observational, we were unable to confirm the causal relationships of these data.

## Conclusions

In summary, this meta-analysis of observational studies indicates that excessive GWG in accordance with the IOM recommendations is associated with the risk of HDP and should therefore be avoided. Additional studies are needed to assess whether this result is causal or reflects a common cause. Importantly, even a modest risk would have significant public health implications. Therefore, high-quality confirmatory studies and appropriate intervention studies are needed to reduce the risks of excessive GWG.

## Additional files

Additional files 1: **Table S1.** Characteristics of the included studies. (DOCX 37 kb)

Additional files 2: **Figure S1.** Sensitivity analyses for the pooled crude data of the cohort studies of GWG above the IOM recommendations. (TIF 6555 kb)

Additional files 3: **Figure S2.** Sensitivity analyses for the pooled crude data of the cohort studies of GWG above the IOM recommendations among women with pregestational normal weight. (TIF 8326 kb)

Additional files 4: **Figure S3.** Funnel plot of the random-effects model showing the pooled estimate of the odds of GWG above the IOM recommendations. (TIF 18910 kb)

## Abbreviations

BMI: Body mass index
CI: Confidence intervals
GH: Gestational hypertension
GWG: Gestational weight gain
HDPs: Hypertensive disorders of pregnancy
IOM: Institute of Medicine
MOOSE: Meta-Analysis of Observational Studies in Epidemiology
OR: Odds ratios
PE: Preeclampsia
WHO: World Health Organization
