# Supplementary material for: Excessive gestational weight gain in accordance with the IOM criteria and the risk of hypertensive disorders of pregnancy: a meta-analysis
Source: BMC Pregnancy Childbirth. 2018 Jul 4;18:281. doi: 10.1186/s12884-018-1922-y (PMC6030787; doi:10.1186/s12884-018-1922-y)
Supplement: Supplementary file 1 — Table S1. Characteristics of the included studies. (DOCX 37 kb) [file 12884_2018_1922_MOESM1_ESM.docx]

**Table S1** Characteristics of the included studies

| Author, year, country | Study design | Sample size | Definition of GWG* | Prepregnancy weight | Definition of outcomes | Inclusion criteria | Exclusion criteria |
| --- | --- | --- | --- | --- | --- | --- | --- |
| Hung, 2016, Taiwan^[^[^15^](#_ENREF_15)^]^ | Retrospective cohort study | 10,973 | WD-PPW | Self-report | Preeclampsia was defined as the combination of hypertension and proteinuria (≥ 300 mg of protein in a 24-hour urine sample) occurring after 20 weeks of gestation. | Women with a live singleton delivered after 37 weeks of gestation | Pregnancies complicated by multiple gestations, fetal chromosomal or structural anomalies, and fetal demise; with pregestational diabetes mellitus and chronic hypertension |
| Tanaka, 2014, Japan^[^[^16^](#_ENREF_16)^]^ | Retrospective cohort study | 1,883 | WD-PPW | Self-report | PIH was defined as a systolic blood pressure of 140 mmHg or more, or a diastolic blood pressure of 90 mmHg or more, on at least two occasions at least 4 h apart in a patient who was normotensive prior to 20 weeks of gestation. |  | Women with multiple gestations, underlying diseases or complications and preterm birth as well as those who were not Japanese |
| Liu, 2015, China^[^[^17^](#_ENREF_17)^]^ | Retrospective analysis of data collected prospectively | 2,973 | WD-PPW | Self-report | GHT was defined as the development of arterial hypertension (as a new phenomenon) in a pregnant woman after 20 weeks of gestation, without enough proteinuria, symptoms or laboratory abnormalities to be classified as preeclampsia. Preeclampsia was defined as the combination of hypertension and proteinuria (≥ 300 mg of protein in a 24-hour urine sample) occurring after 20 weeks of gestation. | Nulliparous women who delivered single live infants | Histories of hypertension, diabetes, heart disease, hepatitis, chronic renal disease or other systemic diseases; a history of drug use before or during pregnancy; a history of alcohol use before or during pregnancy; or a history of smoking before or during pregnancy |
| Li C,2015, China^[^[^18^](#_ENREF_18)^]^ | Retrospective Cohort Study | 48,867 | WD-WEP | Measure | Pregnancy-induced hypertension was diagnosed by a systolic blood pressure of ≥140 mmHg or a diastolic blood pressure of ≥90 mmHg that developed after 20 weeks of gestation and returned to normal within 12 weeks postpartum. | Healthy Han nulliparous women having singleton and full-term births | Multiple gestations, diagnosed diabetes or chronic hypertension; women < 18 years old |
| Enomoto, 2016, Japan^[^[^19^](#_ENREF_19)^]^ | Retrospective Cohort Study | 97,157 | Not mentioned | Not mentioned | PIH was defined as a case in which hypertension (systolic blood pressure ≥140 mmHg, diastolic blood pressure ≥90 mmHg or both) developed after 20 weeks of gestation. | Singleton pregnancies | Women with concomitant hypertension or diabetes as the underlying disease, with a history of cervical conization, who delivered a newborn with congenital anomalies |
| Chung, 2013, Australia^[^[^20^](#_ENREF_20)^]^ | Prospective cohort study | 1,950 | WD-WEP | Measure | Gestational hypertension defined as sBP≥140 mmHg, dBP≥90 mmHg or both on at least 2 occasions 4 h apart after 20 weeks of gestation but before the onset of labor). Preeclampsia was defined as gestational hypertension plus proteinuria ≥300 mg/24 h or spot urine protein: creatinine ratio ≥30 mg/mmol creatinine or urine dipstick protein ≥++ or any multi-system disease. | Healthy, nulliparous women with singleton pregnancies | Ineligible status post recruitment, miscarriage or termination 15-19 weeks, terminations ≥20 weeks due to anomalies or other reasons, underweight BMI prepregnancy |
| Chasan-Taber, 2016, Puerto Rican^[^[^21^](#_ENREF_21)^]^ | A secondary analysis of a multicenter, placebo-controlled randomized double blind trial | 1,293 | WD-PPW | Self-report | Gestational hypertension was defined as two blood pressure measurements greater than 140/90 after 20 weeks of gestation in a previously normotensive woman, with no lab evidence or symptoms of preeclampsia. Preeclampsia was defined as the new onset of hypertension (BP ≥140/90 mmHg) after 20 weeks of gestation in association with proteinuria, either ≥1+ by dipstick or ≥300 mg/24 h in the absence of urinary infection. | Eligibility was restricted to women of Puerto Rican or Dominican Republic heritage (i.e., Caribbean Islanders) | Current medications thought to adversely influence glucose tolerance, multiple gestations; histories of diabetes, hypertension, heart disease or chronic renal disease; and aged younger than 16 years or older than 40 years |
| Johnson, 2013, USA^[^[^22^](#_ENREF_22)^]^ | A secondary analysis of a prospective cohort study | \| 8,293 \| \| --- \| \| | WD-WEP | Measure | Gestational hypertension was defined based on a systolic pressure of greater than or equal to 140 mm Hg or a diastolic pressure of greater than or equal to 90 mm Hg on two separate occasions 2– 240 hours apart after 20 weeks of gestation in the absence of proteinuria. Preeclampsia was defined as gestational hypertension with either proteinuria, defined as greater than or equal to 300 mg in a 24 hour sample, or, if a 24-hour sample was not available, 2+ or higher on dipstick testing, or a protein:creatinine ratio of greater than or equal to 0.35, pulmonary edema, thrombocytopenia, or eclampsia. | Nulliparous (no previous pregnancy lasting more than 19 6/7 weeks) and carrying a singleton gestation between 9 and 16 weeks | Elevated blood pressure (systolic of 135 mmHg or higher or diastolic of 85 mmHg or higher), proteinuria (300 mg in a 24-hour collection or higher or more than trace protein on a urine dipstick), pregestational diabetes, treatment with antiplatelet or non-steroidal anti-inflammatory drugs, uterine bleeding within the week prior to recruitment, uterine malformation, serious medical condition (e.g., epilepsy), known fetal anomalies or aneuploidy, in vitro fertilization resulting in the current pregnancy, and illicit drug or alcohol abuse, delivered prior to 20 weeks, died prior to delivery, had an abortion, or their infant was found to have a major congenital malformation |
| Hannaford, 2017, USA^[^[^23^](#_ENREF_23)^]^ | Retrospective cohort study | 1,120 | WD-WEP | Measure | PEC was defined using the guidelines from the American College of Obstetricians and Gynecologists (ACOG) and the criteria proposed by the National High Blood Pressure Education Program Working Group report in Pregnancy. | Women with singleton gestations who underwent first trimester aneuploidy screening at our ultrasound (US) unit | Women who carried fetuses with structural or chromosomal anomalies or those who lacked maternal height or weight data |
| Fouelifack, 2015, Cameroon^[^[^24^](#_ENREF_24)^]^ | Retrospective cohort study | 462 | Not mentioned | Not mentioned | Not mentioned | A singleton term delivery | Women with incomplete files, unknown prepregnancy weights, severe physical conditions and those who did not consent; sampling was consecutive |
| Li N, 2013, China^[^[^25^](#_ENREF_25)^]^ | Retrospective cohort study | 33,973 | WD-WEP | Measure | Pregnancy-induced hypertension was diagnosed based on a systolic blood pressure of ≥140 mmHg or a diastolic blood pressure of ≥90 mmHg in the 3rd trimester or the use of antihypertensive drugs. | We collected the records of both mothers and their infants who were born in the central urban districts between June 2009 and May 2011. | Multiple births, stillbirths, multiparous women, and mother-child pairs missing any variables required for this analysis |
| Zhou, 2015, China^[^[^26^](#_ENREF_26)^]^ | Retrospective cohort study | 84,656 | WD-PPW | Self-report | Gestational hypertension was defined as having a maternal systolic blood pressure (SBP) of > 140 mm Hg, a diastolic blood pressure (DBP) of > 90 mmHg or both, measured on 2 occasions separated by at least 6 hours beginning after 20 weeks of gestation. Preeclampsia was defined using the same criteria in conjunction with proteinuria > 300 mg on a 24-hour urine collection or proteinuria of at least 1+ on dipstick testing. | Women without a history of chronic hypertension or cardiovascular disease prior to pregnancy, who lived in the urban area of Wuhan during pregnancy, and who delivered a live singleton newborn without birth defects and a gestational age no less than 28 weeks | We excluded those with any missing values for height, prepregnancy weight, or GWG. To eliminate extreme outliers, the data analyses were also limited to women whose height, weight, and GWG were within 5 standard deviations of the mean. |
| de la Torre L, 2011, USA^[^[^27^](#_ENREF_27)^]^ | Retrospective analysis of data collected prospectively | 7,676 | Not mentioned | Not mentioned | Pregnancy-related hypertension was defined as a positive report of gestational hypertension or preeclampsia documented in the outpatient pregnancy outcome record. | Singleton term deliveries (＞37 weeks) reporting no maternal history of cardiovascular disease, diagnosis of pregnancy-related hypertension, or diabetes at the initiation of outpatient nursing services |  |
