# Supplementary figures and images for: Excessive gestational weight gain in accordance with the IOM criteria and the risk of hypertensive disorders of pregnancy: a meta-analysis
Source: BMC Pregnancy Childbirth. 2018 Jul 4;18:281. doi: 10.1186/s12884-018-1922-y (PMC6030787; doi:10.1186/s12884-018-1922-y)

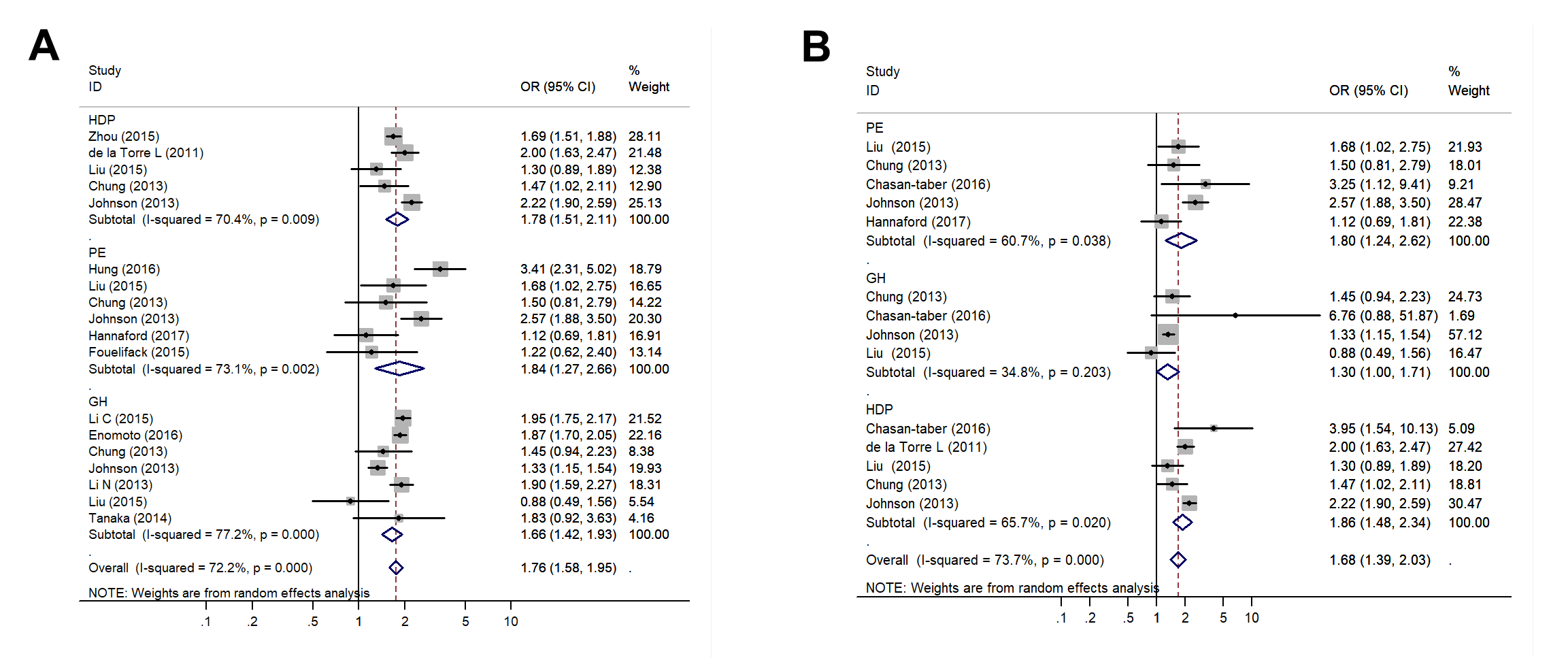

Supplement: Supplementary file 2 — Figure S1. Sensitivity analyses for the pooled crude data of the cohort studies of GWG above the IOM recommendations. (TIF 6555 kb) [file 12884_2018_1922_MOESM2_ESM.tif]

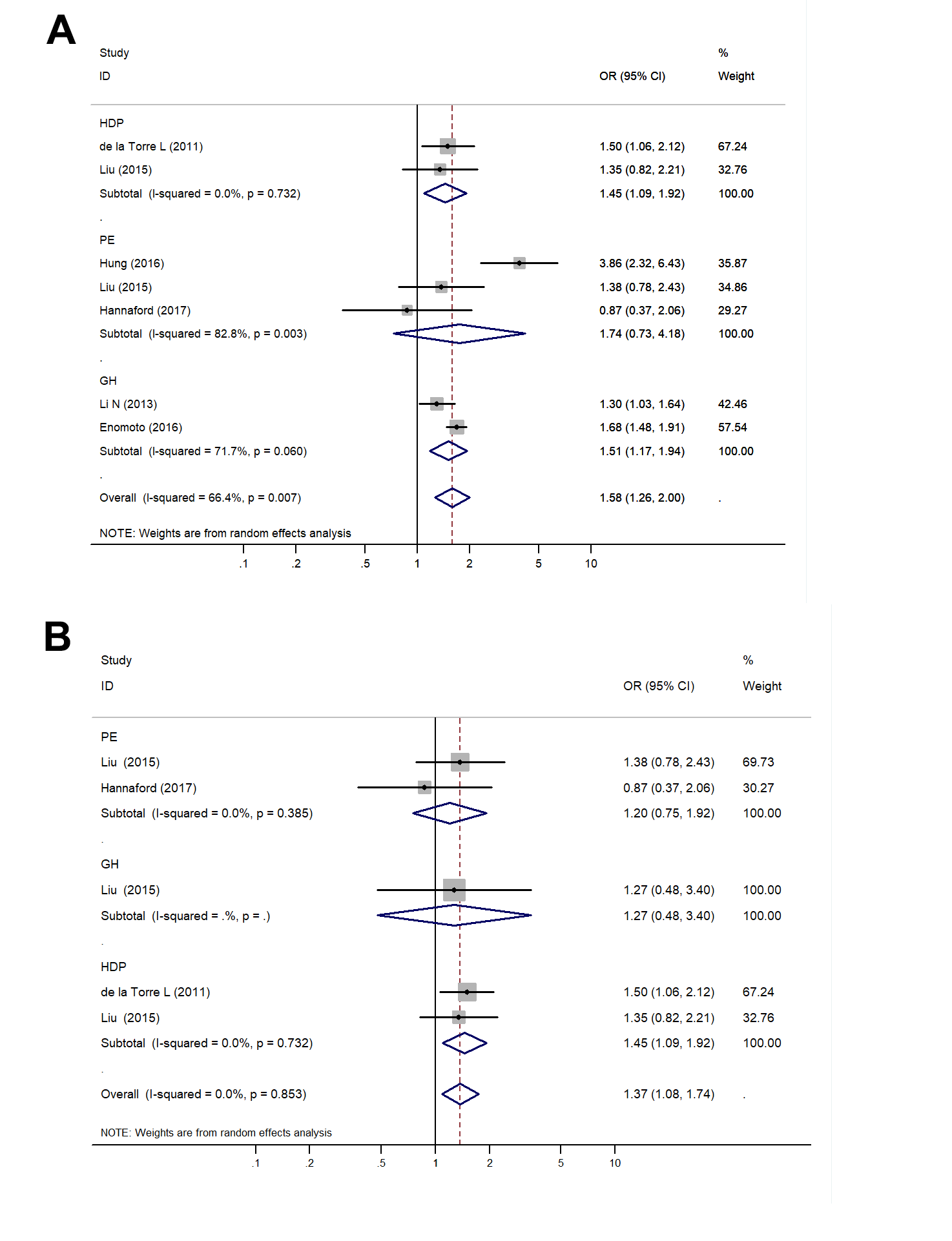

Supplement: Supplementary file 3 — Figure S2. Sensitivity analyses for the pooled crude data of the cohort studies of GWG above the IOM recommendations among women with pregestational normal weight. (TIF 8326 kb) [file 12884_2018_1922_MOESM3_ESM.tif]

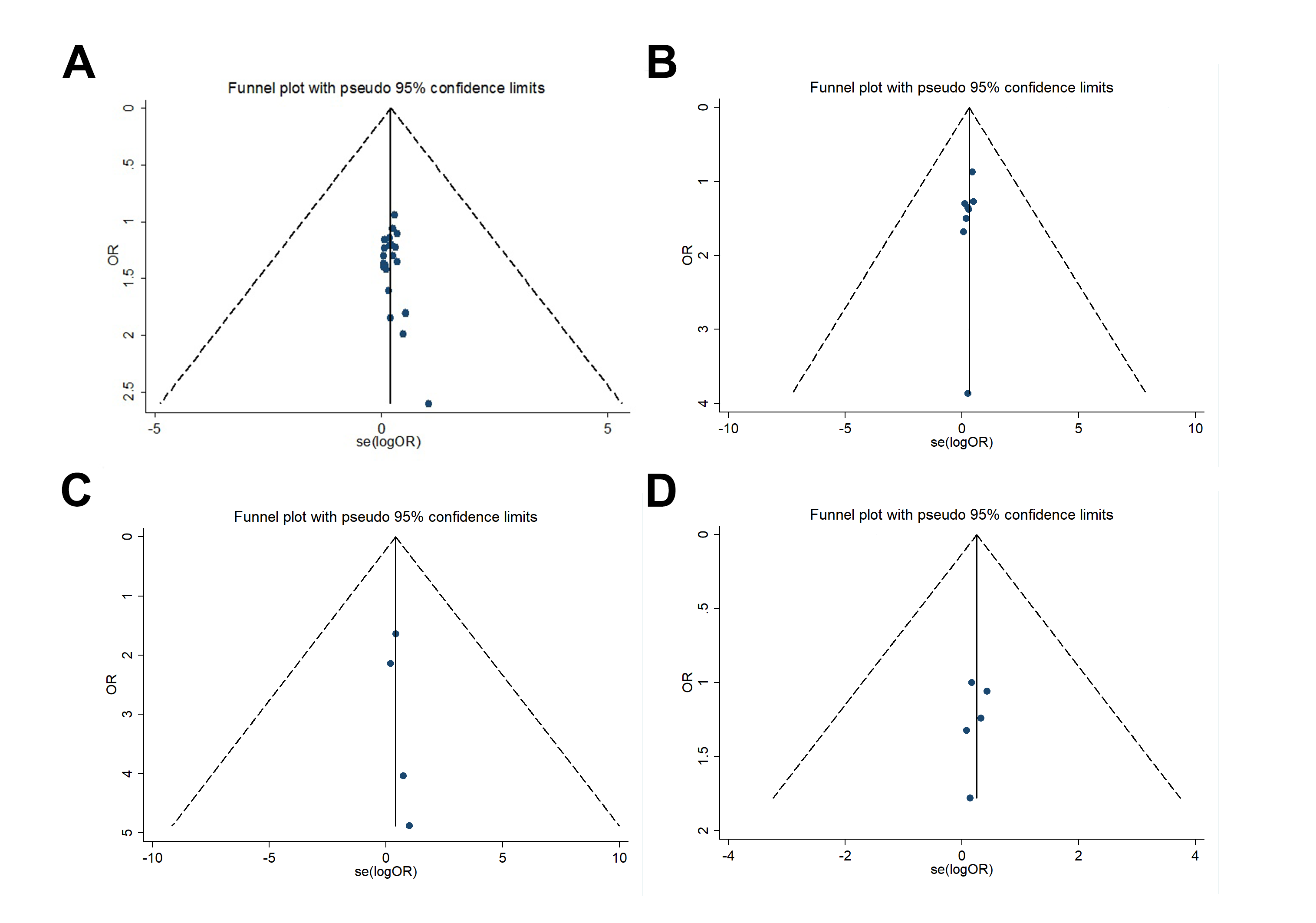

Supplement: Supplementary file 4 — Figure S3. Funnel plot of the random-effects model showing the pooled estimate of the odds of GWG above the IOM recommendations. (TIF 18910 kb) [file 12884_2018_1922_MOESM4_ESM.tif]
